# Epigallocathechin-*O*-3-Gallate Inhibits Trypanothione Reductase of *Leishmania infantum*, Causing Alterations in Redox Balance and Leading to Parasite Death

**DOI:** 10.3389/fcimb.2021.640561

**Published:** 2021-03-25

**Authors:** Job D. F. Inacio, Myslene S. Fonseca, Gabriel Limaverde-Sousa, Ana M. Tomas, Helena Castro, Elmo E. Almeida-Amaral

**Affiliations:** ^1^ Laboratório de Bioquímica de Tripanosomatideos, Instituto Oswaldo Cruz (IOC), Fundação Oswaldo Cruz – FIOCRUZ, Rio de Janeiro, Brazil; ^2^ Laboratório de Esquistossomose Experimental, Instituto Osvaldo Cruz, Fundação Oswaldo Cruz – FIOCRUZ, Rio de Janeiro, Brazil; ^3^ i3S—Instituto de Investigação e Inovação em Saúde, Universidade do Porto, Porto, Portugal; ^4^ ICBAS—Instituto de Ciências Biomédicas Abel Salazar, Universidade do Porto, Porto, Portugal

**Keywords:** EGCG, *Leishmania infantum*, mechanism of action, trypanothione reductase, competitive inhibitor

## Abstract

*Leishmania infantum* is a protozoan parasite that causes a vector borne infectious disease in humans known as visceral leishmaniasis (VL). This pathology, also caused by *L. donovani*, presently impacts the health of 500,000 people worldwide, and is treated with outdated anti-parasitic drugs that suffer from poor treatment regimens, severe side effects, high cost and/or emergence of resistant parasites. In previous works we have disclosed the anti-*Leishmania* activity of (-)-Epigallocatechin 3-*O*-gallate (EGCG), a flavonoid compound present in green tea leaves. To date, the mechanism of action of EGCG against *Leishmania* remains unknown. This work aims to shed new light into the leishmanicidal mode of action of EGCG. Towards this goal, we first confirmed that EGCG inhibits *L. infantum* promastigote proliferation in a concentration-dependent manner. Second, we established that the leishmanicidal effect of EGCG was associated with i) mitochondria depolarization and ii) decreased concentration of intracellular ATP, and iii) increased concentration of intracellular H_2_O_2_. Third, we found that the leishmanicidal effect and the elevated H_2_O_2_ levels induced by of EGCG can be abolished by PEG-catalase, strongly suggesting that this flavonoid kills *L. infantum* promastigotes by disturbing their intracellular redox balance. Finally, we gathered *in silico* and *in vitro* evidence that EGCG binds to trypanothione reductase (TR), a central enzyme of the redox homeostasis of *Leishmania*, acting as a competitive inhibitor of its trypanothione substrate.

## Introduction

Visceral leishmaniasis (VL) is a neglected tropical disease caused by *Leishmania infantum* and *L. donovani* that affects 500,000 people and is fatal in over 95% of cases if left untreated. It is estimated that 50,000 to 90,000 new VL cases occur worldwide each year (World Health Organization, 2018). The disease has a high prevalence in the Americas, particularly in Brazil where 96% of the cases are reported (Pan American Health Organization, 2018). In the absence of a vaccine, VL treatment is largely based on chemotherapy, pentavalent antimonials and amphotericin B being the most used drugs. However, even though these treatments have saved thousands of lives, they can only be administered parenterally, present severe side effects and are expensive (Barral et al., 1991; Grimaldi and Tesh, 1993; Croft and Coombs, 2003; Amato et al., 2008). The introduction of miltefosine in 2014 as another first-line option for VL therapy and the first orally delivered drug, aimed at attending some of those drawbacks (McGwire and Satoskar, 2014). Nevertheless, an increasing number of clinical relapses has meanwhile been reported (Rijal et al., 2013) rendering the development of new, more effective, safer, and easily accessible drugs extremely urgent (Chappuis et al., 2007; Capela et al., 2019).

Flavonoids are natural products that have demonstrated significant antiprotozoal activities (Tasdemir et al., 2006; Gervazoni et al., 2020). Within this chemotype, (-)-Epigallocatechin 3-*O*-gallate (EGCG), the most abundant flavonoid constituent of green tea, was recently demonstrated to display activity against different species of *Leishmania*, including visceral leishmaniasis species (*L. infantum* (Inacio et al., 2019)).

Indeed, we found EGCG to be active *in vitro*, against intracellular amastigotes with a selectivity index over 100, and *in vivo*, in a murine model of infection also without evident toxicity towards treated animals (Inacio et al., 2019). These data and the fact that EGCG can be orally administered encourage further studies towards the potential of this compound for VL therapy. In this regard, one important aspect refers to the characterization of the underlying mechanism of activity. Based on the observation that in mice EGCG can modulate two important antioxidant proteins, hepatic glutathione peroxidase and glutathione reductase (Dong et al., 2016), we considered here the possibility that EGCG activity in *Leishmania* interferes with the parasite antioxidant mechanisms

In *Leishmania* spp. and related trypanosomatid organisms, redox balance is largely accomplished by the enzyme trypanothione reductase (Leroux and Krauth-Siegel, 2016). This molecule functions as a FAD-dependent disulfide oxidoreductase that catalyzes the reduction of trypanothione [*N* ^1^,*N* ^8^-bis-glutathionylspermidine or T(SH)_2_] at expenses of NADPH. Trypanothione is a dithiol, composed of two molecules of glutathione linked by one spermidine bridge. It is the major thiol in trypanosomatids, where it acts as electron donor for a multitude of biological reactions (including the elimination of hydroperoxides), and thus assumes the functions of glutathione in other organisms. The recycling of trypanothione back to its reduced, active form is carried out by the enzyme trypanothione reductase (TR). This process is absolutely critical for parasite redox balance and overall cell viability, as demonstrated by the essential character of TR (Dumas et al., 1997; Tovar et al., 1998). The nearest mammalian homologue of TR is the enzyme glutathione reductase (GR). Despite their overall similarity, TR and GR differ significantly in their thiol-binding sites, making it feasible to target TR with chemical entities that do not compromise GR activity (Castro et al., 2018). Overall, the essential role played by TR in *Leishmania* spp. and its absence from the human host render this molecule an attractive target for the development of new potential drugs.

The present study investigates the mechanism of action of EGCG in promastigotes of *L. infantum*. It confirms that EGCG inhibits promastigote proliferation in a concentration-dependent manner and describes that this phenomenon is associated with mitochondrial depolarization, decreased intracellular ATP concentration, and increased levels of H_2_O_2_. Importantly, it establishes that the leishmanicidal effect of EGCG is completely abolished by PEG-catalase, suggesting that this flavonoid induces an oxidative stress status in promastigotes that culminates in their death. Aligned with this hypothesis, this report presents evidence that EGCG interacts with TR *in silico* and that it acts as a competitive inhibitor for trypanothione binding to TR in *in vitro* enzymatic assays. Overall, this report contributes to clarify the mode of leishmanicidal action of EGCG.

## Materials and Methods

### Reagents

(−)-Epigallocatechin 3-*O*-gallate (molecular formula: C_22_H_18_O_11_; molecular weight: 458.37 g/mol; purity ≥95%), trypanothione, JC-1, horseradish peroxidase (HRP), digitonin, NADP^+^, NADPH, DTNB, FCCP, HEPES, Schneider’s Drosophila medium, fetal calf serum and RPMI 1640 medium were obtained from Sigma Aldrich (St Louis, MO, USA). Amplex Red, and alamarBlue were obtained from Invitrogen Molecular Probes (Leiden, The Netherlands). Other reagents were purchased from Merck (Sao Paulo, Brazil). Deionized distilled water was obtained using a Milli-Q system of resins (Millipore Corp., Bedford, MA, USA) and was used to prepare all solutions.

### Parasites

The MHOM/MA/67/ITMAP263 strain of *Leishmania infantum* was used throughout this study. *Leishmania infantum* amastigotes were isolated from BALB/c mice and maintained as promastigotes at 26°C in Schneider’s medium supplemented with 20% fetal bovine serum (v/v), 100 U/ml penicillin and 100 μg/ml streptomycin. Parasite maintenance was achieved by passaging every 3 days. Female BALB/c mice (8−10 weeks) were provided by the Instituto Ciências e Tecnologia em Biomodelos (ICTB/FIOCRUZ). All animals were bred and maintained at the Instituto Oswaldo Cruz according to Guide for the Care and Use of Laboratory Animals of the Brazilian National Council for Control of Animal Experimentation (CONCEA). The protocol was approved by the Committee on the Ethics of Animal Experiments of the Instituto Oswaldo Cruz (CEUA-IOC, License Number: L-11/2017).

### Promastigote Proliferation Assay

*L. infantum* (10^6^/mL) promastigotes were incubated with different concentrations of EGCG (0.015–1 mM) or vehicle (PBS) for 72 h. The cell density was estimated with the addition of alamarBlue (10% v/v) for 3 h at 26°C. The absorbance was measured at 570 nm using a spectrophotometer. The growth curve was initiated with 1 x 10^6^ cells/ml. The 50% inhibitory concentration (IC_50_) was determined by logarithmic regression analysis using GraphPad Prism 6 (GraphPad Software, La Jolla, CA, USA). The experiments were run three times in triplicate.

### Hydrogen Peroxide Measurement

Hydrogen peroxide production was measured using Amplex Red and horseradish peroxidase (HRP) (Fonseca-Silva et al., 2015). Promastigotes were treated for 72 h in the absence or presence of EGCG (125, 250 and 500 µM). Cells were harvested and resuspended in Hanks balanced salt solution (HBSS). The cell number was obtained by counting using a Neubauer chamber. Promastigotes (2x10^7^ cells/mL) were incubated with HBSS containing 10 μM Amplex Red reagent, 64 μM digitonin (to permeabilize parasites) and 10 U/ml HRP for 30 min at 26°C. Fluorescence was monitored at excitation and emission wavelengths of 560 and 590 nm, respectively, in a spectrofluorometer. H_2_O_2_ concentrations were calculated from an H_2_O_2_ standard curve.

### Mitochondrial Membrane Potential (ΔΨ_m_) Assay

The cationic probe JC-1 was used to determine the mitochondrial membrane potential (ΔΨ_m_) as described previously (Fonseca-Silva et al., 2011). Promastigotes (10^6^ cells/ml) were cultured for 72 h in the absence or presence of EGCG (125 – 500 µM). Cells were harvested and resuspended in HBSS. The cell number was obtained by counting in a Neubauer chamber. Promastigotes (10^7^ cells/ml) were incubated with JC-1 (10 μg/ml) for 10 minutes at 37°C. After washing twice with HBSS, fluorescence was measured using a spectrofluorometer at 530 nm and 590 nm using an excitation wavelength of 480 nm. The ratio of the values obtained at 590 nm and 530 nm was plotted as the relative ΔΨ_m_. The mitochondrial uncoupling agent carbonyl cyanide *p*-trifluoromethoxyphenylhydrazone (FCCP; 20 μM) was used as control.

### Determination of Intracellular ATP Concentration

Intracellular ATP concentrations in *L. infantum* promastigotes were measured using a CellTiter-Glo luminescent assay (Promega), where the signal was proportional to ATP concentration. Briefly, promastigotes were treated for 72 h in the absence or presence of EGCG (125 – 500 μM). Cultures were washed three times, and parasite concentration was adjusted to 10^7^ cells in 200 μl of PBS. A 50-µl aliquot of each sample was transferred to a 96-well plate and mixed with the same volume of CellTiter-Glo. Plates were incubated in the dark for 10 min, and bioluminescence was measured using a GloMax-Multi Microplate Multimode Reader (Promega). ATP concentrations were calculated from the ATP standard curve.

### Docking Studies

The crystal structures of oxidized and reduced TR (PDB 2JK6 and 4ADW, respectively) were downloaded from the RCSB Protein DataBank (www.rcsb.org) (Berman et al., 2000). The atomic partial charges were calculated in the PDB2PQR Server (www.nbcr-222.ucsd.edu) using the AMBER forcefield, and the protonation state was determined using PROKA at pH 7.4. All proteins were converted to PDBQT format using Autodock Tools. A cubic grid map with 80 Å of side and spacing of 0.375 Å was built centralized on active site residues (x, y, z coordinates: 23.3, 51.9, -15.6) of TR on both states (oxidized and reduced) using Autodock Tools. (Baiocco et al., 2009a; Baiocco et al., 2009b; Venkatesan et al., 2010; Colotti et al., 2013).

The docking of EGCG to *L. infantum* TR was performed with AutoDock (Morris et al., 2009). The grid box parameters for both states of TR were center x/y/z, 23.3/51.9/-15.6, dimension x/y/z, 80/80/80 and spacing, 0.375 Å (Ortalli et al., 2018). Grid boxes were set around the active site residues involved in the biological functions of the protein used in this study (Baiocco et al., 2009a; Baiocco et al., 2009b; Venkatesan et al., 2010; Colotti et al., 2013).

The docking procedure was performed using 500 runs of Lamarckian Genetic Algorithm (LGA). Ligand binding position and interaction analysis were performed using PyMOL software and the 2D interaction was visualized with BIOVIA Discovery Studio 2020 (Dassault Systems BIOVIA, Discovery Studio Modeling Environment, Release 2017, San Diego: Dassault Systems, 2016).

### Heterologous Expression and Purification of Recombinant *Li*TR

Full-length *Li*TR (tritryp ID: LINF_050008500) was PCR-amplified from the genomic DNA of *L. infantum* and cloned into the NdeI/XhoI sites of the pET23a(+) plasmid. The resulting construct was introduced in *Escherichia coli* BL21(DE3) for expression of a modified version of *Li*TR containing a carboxy-terminal six-histidine tag (LiTR.6His), as follows. Bacteria were grown at 37 °C, in Luria Bertani (LB) medium supplemented with 50 μg/ml ampicillin and 0.1% (w/v) glucose, until an OD_600nm_ of 0.6. *Li*TR.6His expression was induced by addition of 2 mM isopropyl-β-D-thiogalactopyranoside (IPTG) and further incubation at 37 °C, for 4 h. Bacteria were pelleted and suspended in 20 mM Tris-HCl pH7.6, 500 mM NaCl, 10 mM imidazole, at a ratio of 1 g wet weight per 5 ml buffer, followed by disruption by sonication and centrifugation at 30,000 ×g, for 20 min, at 4 °C. *Li*TR.6His was obtained as a soluble protein in the supernatant of lysed bacteria and purified by passage through a 1 ml HisTrap column (GEHealthcare), followed by elution in a 25-500 mM imidazole gradient, at 4 °C and a flow rate of 0.5 ml/min, using an ÄKTAprime plus equipment (GEHealthcare). Fractions confirmed by SDS-PAGE to contain *Li*TR.6His were pooled, passed through a PD-10 desalting column packed with Sephadex G-25 resin (GEHealthcare), and recovered in 50 mM HEPES pH 7.6, 1 mM EDTA. *Li*TR.6His quality and purity was confirmed by SDS-PAGE, and its concentration estimated using the bicinchoninic acid assay (Pierce) with bovine serum albumin as standard.

### Trypanothione Reductase Activity Assay

This assay, in which generation of reduced trypanothione [T(SH)_2_] by TR is coupled with the chemical oxidant 5,5’-dithiobis-(2-nitrobenzoic acid) (DTNB; or Ellman’s reagent) (Hamilton et al., 2003; Richardson et al., 2009), was performed in 96-well plates. Reaction mixtures contained 50 mM HEPES pH 8.0, 1 mM EDTA, 30 µM NADP^+^, 1 µM oxidized trypanothione (TS_2_), 0.1 mM DTNB, 0.15 mM NADPH and increasing concentrations of EGCG (7.8 – 500 µM). Reactions were started by addition of 7 nM recombinant *Li*TR.6His (*i.e.* C-terminally histidine-tagged *L. infantum* TR), purified from *Escherichia coli* by nickel chromatography and quantified by the bicinchoninic acid (BCA) protein assay (Pierce), using bovine serum albumin (BSA) as standard. Upon addition of *Li*TR.6His, the rate of TNB formation was monitored at 410 nm at 25°C for 5 minutes using a UV-2401PC UV–vis recording spectrophotometer (Shimadzu Scientific).

### Determination of Kinetic Parameters

Kinetics assays were performed in 96-well plates, following the DTNB-coupled reduction of trypanothione, as described above. Reactions were carried out under a constant concentration of *Li*TR.6His (0.7 nM) and different concentrations of EGCG (100 and 200 µM) and of trypanothione (2, 4, 6, 8, 12, 21, 30 and 60 µM). First, a reaction mixture containing trypanothione, EGCG and 15 mM NADPH was prepared. Next, the reaction was started with addition of a mixture containing 50 mM HEPES pH 8.0, 1 mM EDTA, 8.5 µM NADP^+^, 25 µM DTNB and 0.7 nM TR. The rate of TNB formation was monitored at 410 nm at 25°C for 5 minutes using a UV-2401PC UV–vis recording spectrophotometer (Shimadzu Scientific). The kinetic parameters of *K_m_* and *V_max_* were calculated using a computerized nonlinear regression fit of the data obtained to the Michaelis–Menten equation. The resulting data were also analyzed under Lineweaver–Burk plots.

### Statistical Analysis

All experiments were performed in three independent trials performed in triplicate. Data were analyzed using the Mann-Whitney test or one-way analysis of variance (ANOVA) followed by Tukey’s posttest in GraphPad Prism 6 (GraphPad Software). Differences were considered statistically significant at p ≤ 0.05. Data are expressed as means ± standard error.

## Results

### EGCG Impairs Survival of *L. infantum* Promastigotes

**Figure 1:**
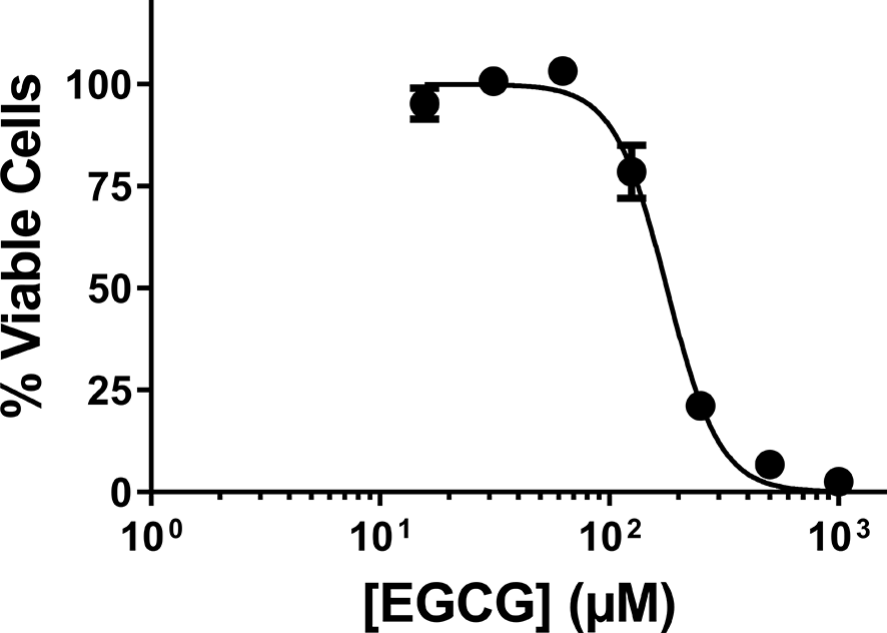
Effect of EGCG on *Leishmania infantum*. Promastigotes of *L. infantum* were cultivated in Schneider’s Drosophila medium at 26°C for 72 h in the absence or presence of EGCG (0.015 – 1 mM). Cell viability was measured using the alamar Blue assay. In the control (absence of EGCG), the same volume of PBS (solvent of EGCG) was added to the growth medium of controls. Values represent mean ± standard error of three different experiments.

Prior to this work, EGCG has been acknowledged for its cytotoxicity against *Leishmania* promastigotes of different species (Inacio et al., 2012; Inacio et al., 2014). Here, we expanded on those findings, towards the calculation of the IC_50_ of EGCG against promastigotes of *L. infantum*. To accomplish this, we exposed parasites to different concentrations of the flavonoid (0.015 − 1 mM) for 72 h, after which time we assessed cellular viability by the alamar Blue assay. The results of this analysis, plotted in **Figure 1**, show that EGCG inhibits promastigote survival in a concentration-dependent manner, reaching 97.5% inhibition at the highest concentration tested (1 mM) (**Figure 1**). Logarithmic regression (R-square = 0.9806) of this concentration-dependent curve revealed an IC_50_ of 177.9 µM.

### EGCG Leads to Mitochondria Depolarization and Impairs ATP Production

**Figure 2:**
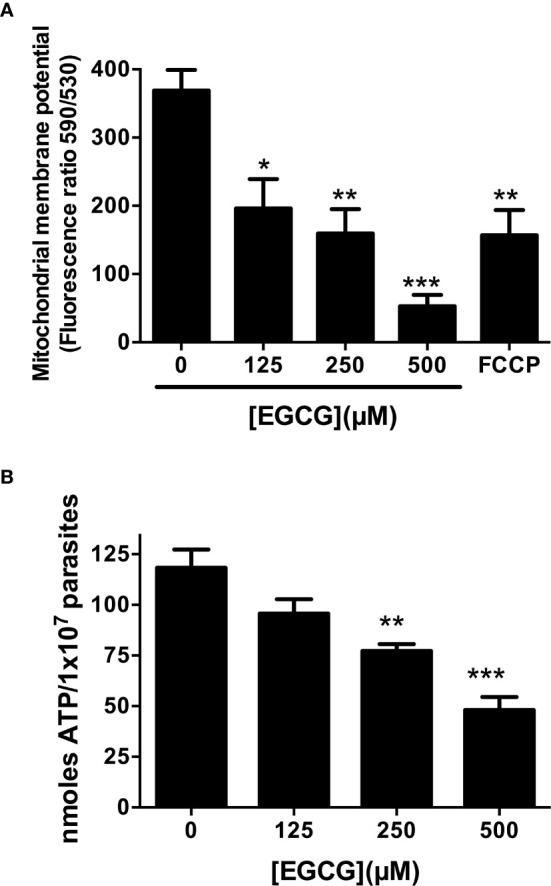
EGCG induces mitochondrial depolarization in *L. infantum* with concomitant loss of intracellular ATP. *Leishmania infantum* promastigotes were cultivated in Schneider’s Drosophila medium at 26°C for 72 h in the absence or presence of 125 – 500 µM EGCG. Promastigotes were stained with the potentiometric probe JC-1 (10 µg/ml). The positive control was treated with FCCP (20 µM) for 20 minutes. In the control (absence of EGCG), the same volume of vehicle (PBS) was added to the growth medium. Concentration-dependent alterations in relative mitochondrial membrane potential (ΔΨ_m_) values are expressed as the ratio of the fluorescence measurements at 590 nm (for J-aggregate) versus 530 nm (for J-monomer) **(A)**. Promastigotes were incubated with EGCG (125 - 500 µM) for 72 h. Intracellular ATP concentrations were measured using a bioluminescence assay. The results are expressed as ATP concentration **(B)**. Data represent means ± standard errors of three different experiments run in triplicate. * indicates a significant difference relative to the control group (p < 0.05); ** indicates a significant difference relative to the control group (p < 0.01); *** indicates a significant difference relative to the control group (p < 0.001).

EGCG induces mitochondrial membrane potential (ΔΨ_m_) depolarization in *Leishmania braziliensis* and *L. amazonensis* (Inacio et al., 2012; Inacio et al., 2014). To characterize the deadly phenotype imposed by EGCG on *L. infantum* promastigotes, we set out to evaluate the mitochondrial membrane potential (ΔΨ_m_) of parasites treated with this flavonoid compound (125, 250 and 500 µM) for 72 h. Resorting to the fluorometric JC-1 probe assay, we observed that EGCG induces a concentration-dependent decrease in the ΔΨ_m_, achieving a reduction of 85.6% upon treatment with 500 µM EGCG (**Figure 2A**). These results indicate that EGCG treatment impacts on promastigote mitochondrial function.

Depolarization of mitochondrial membrane usually leads to a decrease in ATP synthesis, resulting in parasite death (Inacio et al., 2014; Kaplum et al., 2016; Teixeira de Macedo Silva et al., 2018). Accordingly, we evaluated intracellular ATP concentrations in EGCG-treated *L. infantum* promastigotes. As shown in **Figure 2B**, EGCG reduces intracellular ATP levels in a concentration-dependent manner, reaching a reduction of 59.3% in promastigotes treated with 500 µM EGCG for 72 h.

A negative correlation was observed between the percent inhibition of *L. infantum* promastigotes and both ΔΨ_m_ depolarization (Pearson correlation r = -0.9025) and reduction of intracellular ATP levels (Pearson correlation r = -0.9506), suggesting that these two phenomena are directly associated to EGCG-induced parasite death.

### EGCG Exerts Its Leishmanicidal Action by Means of a H_2_O_2_ Boost

**Figure 3:**
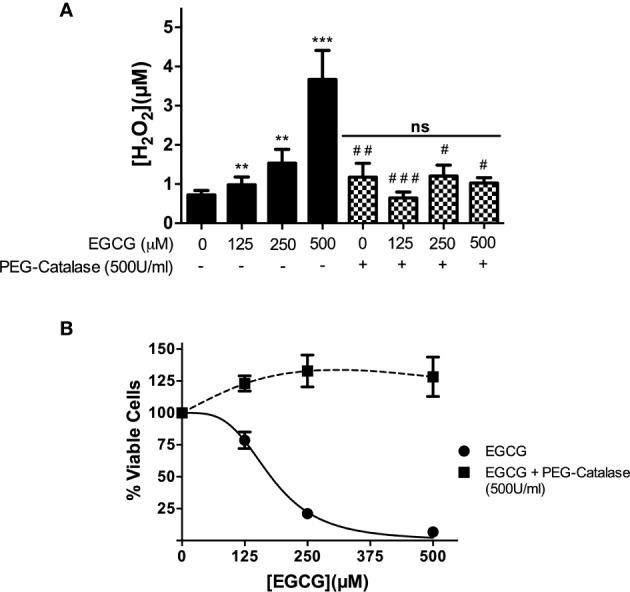
EGCG induces a boost of H_2_O_2_ in *L. infantum* promastigotes that leads to parasite death. Promastigotes of *L. infantum* were cultivated in Schneider’s Drosophila medium at 26°C for 72 h with EGCG (125–500 µM) in the absence or in the presence of PEG-catalase (500 U/ml). H_2_O_2_ was measured with Amplex Red and expressed as the H_2_O_2_ concentration **(A)**. Cellular viability was measured using the alamar Blue assay **(B)**. Values represent mean ± standard error of three different experiments. ** indicates a significant difference relative to the control group (p < 0.01); *** indicates a significant difference relative to the control group (p < 0.001); # indicates a significant difference relative to the *L. infantum* incubated with 500 µM of EGCG (p < 0.05); ## indicates a significant difference relative to the *L. infantum* incubated with 500 µM of EGCG (p < 0.01); ### indicates a significant difference relative to the *L. infantum* incubated with 500 µM of EGCG (p < 0.001).

EGCG is known to increase hydrogen peroxide (H_2_O_2_) concentration in different cell types (Inacio et al., 2014; Kim et al., 2014). To investigate whether synthesis of this oxidant also occurred in promastigotes, we monitored its levels of this oxidant in EGCG-treated parasites (125 – 500 µM) during 72 h resorting to Amplex Red. The outcome of this analysis, depicted in **Figure 3**, revealed that the amount of the hydroperoxide increases with increasing doses of EGCG. Illustrating this, the concentration of H_2_O_2_ increased 5.1-fold in promastigotes treated with 500 µM EGCG, relative to non-treated controls (**Figure 3A**). In short, these data show that exposure to EGCG leads to H_2_O_2_ accumulation in *L. infantum* promastigotes.

Next, we moved on to investigate whether the leishmanicidal activity of EGCG is mediated by the H_2_O_2_ boost. Towards this end, we co-incubated EGCG-treated *L. infantum* promastigotes with polyethylene glycol (PEG)-catalase (500 U/ml), an enzymatic formulation that catalytically decomposes H_2_O_2_. First, we confirmed that PEG-catalase decreases H_2_O_2_ levels in promastigotes treated with EGCG (**Figure 3A**). Second, we observed that PEG-catalase completely abolishes the cytotoxic effect of EGCG towards *L. infantum* promastigotes (**Figure 3B**). In short, these results strongly suggest that EGCG mediates death of *L. infantum* promastigotes by promoting accumulation of H_2_O_2_ to levels that are not tolerated by parasites.

### In Silico Docking Analysis Sustains EGCG Interaction With Trypanothione Reductase

Our observation that EGCG triggers a H_2_O_2_ hike in *L. infantum*, prompted us to investigate whether this phenomenon occurs *via* inactivation of the enzymatic machinery responsible for hydroperoxide elimination, which in *Leishmania* spp. is centralized in trypanothione reductase (TR). We started by performing *in silico* docking studies between EGCG and TR, looking for theoretical thermodynamic and *K_i_* values characterizing the interaction between these molecules. Docking analysis was conducted using two crystalized forms of TR (TR_ox_ oxidized; PDB: 2JK6 and TR_red_ reduced; PDB: 4ADW) which differ in what concerns the oxidation state of trypanothione (Baiocco et al., 2009a; Baiocco et al., 2009b; Colotti et al., 2013). Molecular docking analysis of EGCG with either TR_ox_ or TR_red_ yielded a total of 500 poses of potential interactions, which we grouped in clusters according to conformation similarity [root-mean-square deviation (RMSD) < 2 Å]: i) the cluster with lowest energy, and ii) the cluster containing the most prevalent conformation.

Looking at the lowest energy cluster, we could predict binding energy (ΔG) values of -7.39 or -7.09 kcal/mol between EGCG and TR_ox_ or TR_red_, respectively, which translated into theoretical *K_i_* values of 3.85 and 6.40 µM (**Table 1**). Interestingly, these values changed for EGCG/TR_red_ interaction in the most prevalent cluster – ΔG increased to -6.4 kcal/mol, accompanying the raise in *K_i_* to 20.5 µM (**Table 1**).

**Table 1:** Docking calculations of interactions of EGCG with oxidized and reduced states of trypanothione reductase (TR).

TR_ox_ (2JK6)		TR_red_ (4ADW)	
**Lower energy**		**Lower energy**	
Binding Affinity (kcal/mol)	-7.39	Binding Affinity (kcal/mol)	-7.09
**Ki**	**3.85 µM**	**Ki**	**6.40 µM**
Rank Cluster/Total Cluster	1/94	Rank Cluster/Total Cluster	1/113
N° poses/Cluster	36	N° poses/Cluster	14
**More prevalence**		**More prevalence**	
Binding energy (kcal/mol)	-7.39	Binding energy (kcal/mol)	-6.40
**Ki**	**3.85 µM**	**Ki**	**20.49 µM**
Rank Cluster/Total Cluster	1/94	Rank Cluster/Total Cluster	3/117
N° poses/Cluster	36	N° poses/Cluster	50

Results of 500 docking runs performed with the AutoDock-based Lamarckian Genetic Algorithm (LGA). Grid map was built around the active site residues of TR on oxidized and reduced states.

From this analysis, we obtained theoretical support to our premise that EGCG interacts with TR. To gain insight into the binding site of EGCG within the TR quaternary structure, we moved on to carry out a detailed structural analysis of EGCG/TR *in silico* models.

### In Silico Structural Analysis Supports EGCG Interaction With the Trypanothione Binding Site of TR

**Figure 4:**
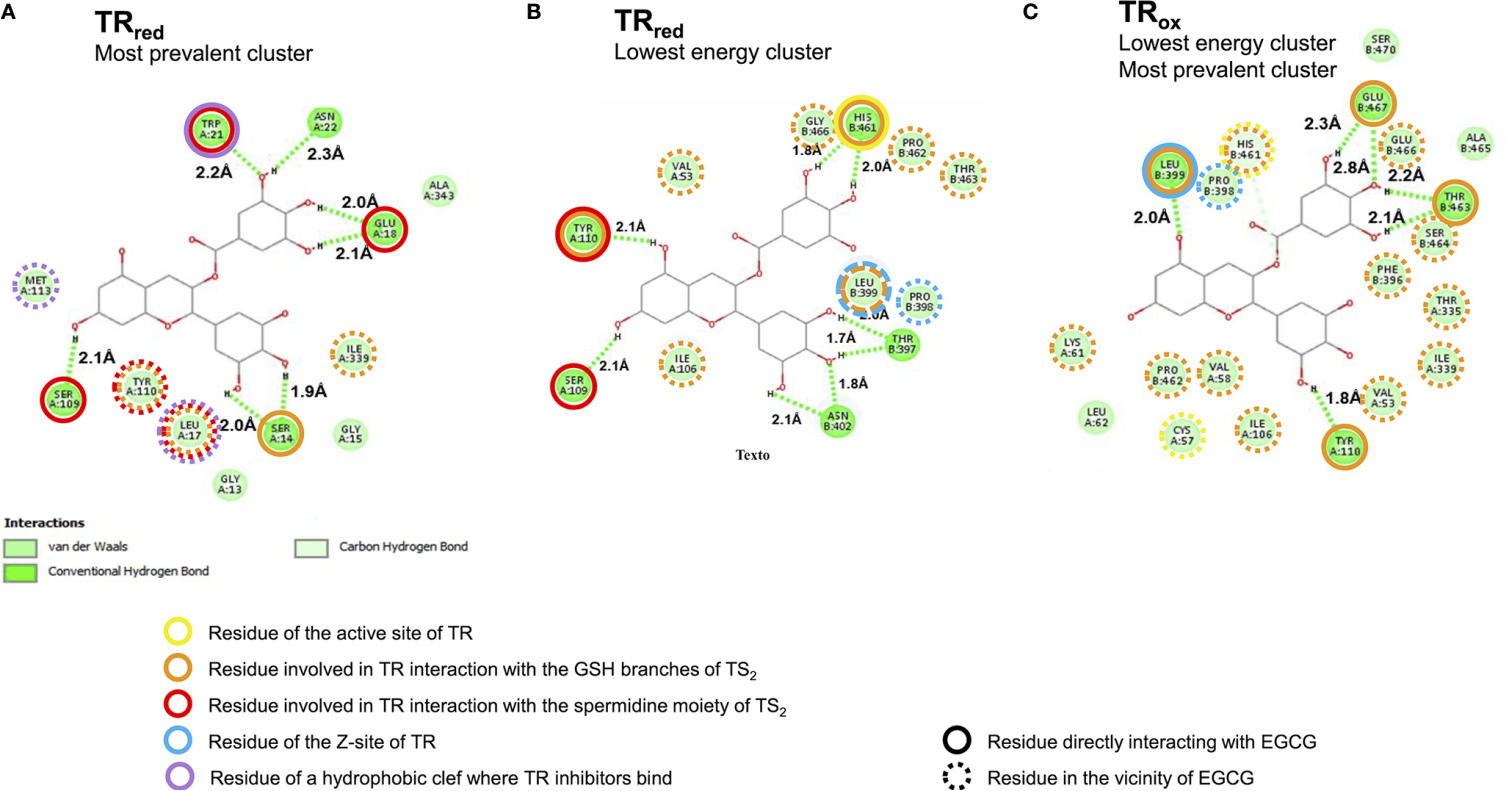
High confidence *in silico* models predict EGCG binding to the trypanothione binding site of trypanothione reductase (TR). Two dimensional representation of the interaction between EGCG (central carbon structure) and TR residues (green circles with aminoacid numbering; the letters A and B refer to different monomers) in **(A)** the most prevalent and also lowest energy EGCG/TR_ox_ cluster, **(B)** the lowest energy EGCG/TR_red_ cluster, and **(C)** the most prevalent EGCG/TR_red_ cluster.

Trypanothione reductase is a homodimeric enzyme with two symmetric active sites, wherein reducing equivalents are conveyed from NADPH to oxidized trypanothione (Baiocco et al., 2009a; Baiocco et al., 2009b; Colotti et al., 2013). Apart from NADPH- and trypanothione-binding pockets, each TR monomer is also comprised of a FAD-binding site and one interface domain. In our *in silico* structural analysis of EGCG/TR interaction, we wondered which of these TR sites are preferentially targeted by EGCG. To assess this, we looked into high confidence molecular models of EGCG interaction with both TR_ox_ and TR_red_, taking into account both the lowest energy and the most prevalent clusters (**Figure 4**). In the case of EGCG/TR_ox_, both clusters yielded similar results, therefore similar interactions (**Figure 4A**).

The results of this *in silico* analysis, depicted in **Figure 4**, predicted that EGCG accommodates to the trypanothione binding site of TR, regardless the redox state of the enzyme (**Figure 4** and **Figure S1**). Indeed, EGCG can potentially establish hydrogen bonds (**Figure 4**, dashed green lines) with residues that directly bind the glutathione (GSH) branches of trypanothione (**Figure 4**, orange circles), and, particularly in the reduced state of TR, also with residues implicated in TR interaction with the spermidine moiety of trypanothione (**Figures 4B, C**, red circles). The preference of EGCG for spermidine-interacting residues is even more striking in the most prevalent EGCG/TR_red_ cluster (**Figure 4C**). By interacting with trypanothione-binding residues, EGCG ends up occupying the trypanothione binding pocket of TR, surrounded by residues that build up this site (**Figure 4**, dashed orange and red circles).

Other EGCG/TR interactions brought into light by this *in silico* analysis include: i) EGCG interaction with residues of the Z-site (**Figures 4B, C**, blue circles), an hydrophobic cavity with interest for drug development owing to its accessibility by hydrophobic compounds with TR-inhibitory activity; and ii) EGCG docking near a hydrophobic wall formed by residues Leu17, Trp21 and Met113 (**Figure 4C**, purple circles), which together form a target region for competitive inhibition by TR inhibitors (Meiering et al., 2005; Baiocco et al., 2013; Colotti et al., 2013; Battista et al., 2020).

From this structural modelling assessment, we conclude that EGCG has the potential to accommodate within the trypanothione binding site of TR and therefore act as a competitor for this substrate.

### *In Vitro* Analyses Confirm That EGCG Is a Competitive Inhibitor of Trypanothione Reductase

To provide experimental demonstration to the *in silico* prediction that EGCG can interact with and inhibit TR by competing with trypanothione, we set out to perform *in vitro* enzymatic assays of TR activity in the presence of this flavonoid. As target enzyme we used recombinant *Li*TR.6His (i.e. *L. infantum* TR with a C-terminal histidine tag), purified from *E. coli*. Our assay consisted in monitoring TR activity based on the DTNB-based colorimetric test originally described by Hamilton et al. (Hamilton et al., 2003). Briefly, this assay combines the TR-catalyzed reduction of TS_2_ to T(SH)_2_, and re-oxidation of the T(SH)_2_ product back to TS_2_ coupled to reduction of DTNB, forming a yellow thionitrobenzoate (TNB) ion. This method maintains a constant substrate concentration, saving on reagents, and enables the linearity of the assay for at least 60 min.

**Figure 5:**
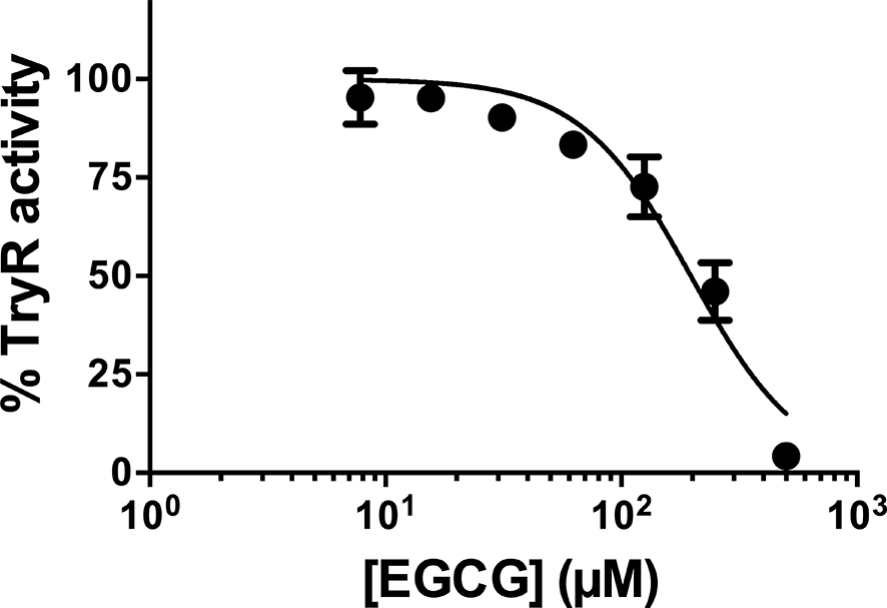
*L. infantum* recombinant TR activity was inhibited by EGCG. *L. infantum* recombinant TR activity was assayed at 25°C and pH 7.5 in the presence of increasing concentrations of EGCG (7.8 – 500 µM), and the assays were carried out in triplicate. Control experiments were carried out in the absence of the inhibitor. Values represent mean ± standard error of three different experiments run in triplicate.

We started by testing the impact that increasing concentrations of EGCG have on TR activity. The results, plotted in **Figure 5**, evidence that, TR activity is inhibited by the flavonoid in a concentration-dependent manner. Almost full inhibition (95.8%) is achieved at the highest concentration of EGCG tested (500 µM). Under these assay conditions, the estimated IC_50_ of EGCG against *Li*TR.6His is 193.8 µM.

**Figure 6:**
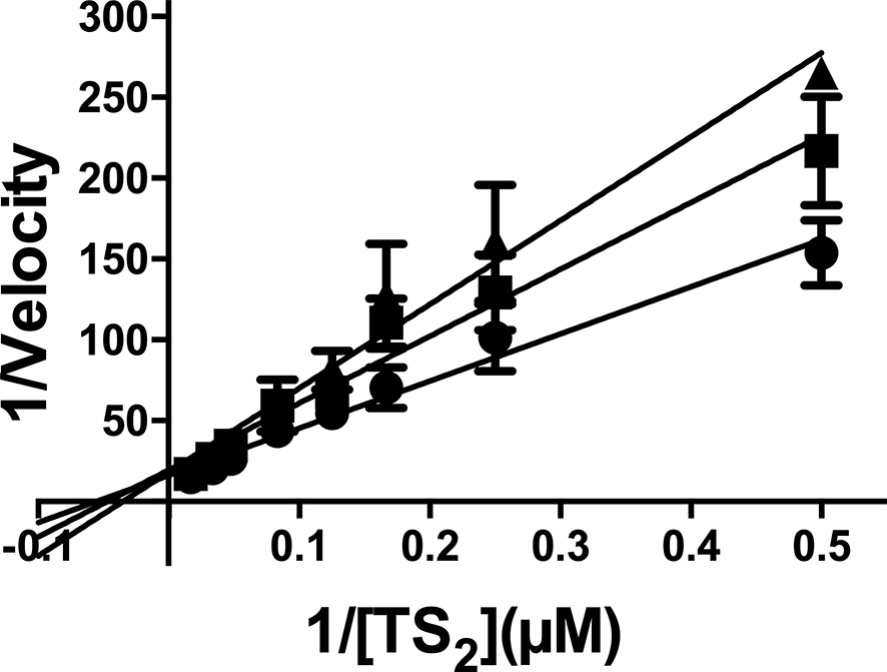
Kinetic analysis of TR activity in the presence of EGCG. The enzyme concentration was maintained as a constant, while the substrate [T(S)_2_] varied as described in the abscissa, and EGCG varied as follows: no addition (closed circles); 100 µM EGCG (closed squares); 200 µM EGCG (closed triangles). The values are presented as the mean ± standard error of three different experiments run in triplicate.

Next, we moved on to characterize the type of inhibition that EGCG exerts over TR. This was done by performing a set of reactions with a fixed amount of *Li*TR.6His and varying concentrations of EGCG (0, 100 and 200 µM) and trypanothione (2 - 60 µM). The velocities estimated for these set of reactions were analyzed in a Lineweaver-Burk plot (1/V vs. 1/[T(SH)_2_]) across the varying concentrations of the inhibitor (**Figure 6**). From this analysis, we obtained several pieces of insightful data. First, results obtained in the absence of ECGC (**Figure 6**, black circles), allowed us to calculate the *K_M_* and *V_max_* values for the trypanothione substrate (36.4 ± 10 µM and 0.11 ± 0.02 nmoles 2TNB.mg^-1^.min^-1^, specifically) (**Table 2**). Second, when the same kinetic parameters were calculated in the presence of 100 and 200 µM EGCG, they revealed invariable *V_max_* values and increasing *K_M_* (60.8 ± 15.9 µM and 66.4 ± 18.5 µM, respectively) (**Table 2**), a behavior that conforms with the hypothesis that EGCG acts as a competitive inhibitor of trypanothione. The *K_i_* value calculated from the Dixon plot analysis is 293.9 ± 66.3 μM (Fig. S2).

**Table 2:** Kinetic parameters of *L. infantum* recombinant trypanothione reductase (TR) in the presence of EGCG.

EGCG (µM)	Kinetic parameters	
	*K_M_* (µM)	*V_max_* (nmoles 2TNB x mg^-1^ x min^-1^)
0	36.4 ± 10.0	0.11 ± 0.02
100	60.8 ± 15.9	0.12 ± 0.02
200	66.4 ± 18.5	0.12 ± 0.02

Values represent means ± standard errors of at least four independent experiments performed in triplicate. The K_M_ and V_max_ of trypanothione were calculated using a computerized nonlinear regression fit of the data to the Michaelis–Menten equation.

## Discussion

Visceral leishmaniasis is a life-threatening form of infection by protozoan *L. infantum* and *L. donovani* parasites, against which new therapeutic formulas are in urgent demand to overcome the limitations of the currently-in-use, leishmanicidal drugs. Natural products, with their enormous chemical diversity, are a major resource for the discovery and development of novel therapeutics (Ioset, 2008; Ndjonka et al., 2013). For parasitic diseases, most of the drugs in use are derived from natural products, often carrying specific modifications to maximize effectiveness (Gervazoni et al., 2020). (−)-Epigallocatechin 3- *O*-gallate (EGCG), the most abundant flavanol constituent of green tea (*Camellia sinensis* (L.) Kuntze; Theaceae), is widely studied and has generated considerable interest as a biologically active compound with a wide range of potential therapeutic activities (Khan and Mukhtar, 2018). In a previous study from our group, EGCG was found to exhibit an IC_50_ of 2.6 µM against intracellular, mammalian-stage *L. infantum* amastigotes, and the ability to reduce *L. infantum* loads in the livers of infected mice in a dose-dependent manner, without compromising the overall health condition of the murine host [consistent with its high Selectivity Index (167.8)] (Inacio et al., 2019). The present report expands on these findings and tries to dig into the mechanistic mode of action of EGCG.

In our study, we started by confirming that, as reported for other *Leishmania* species (Inacio et al., 2012; Inacio et al., 2014), EGCG is active against extracellular, insect stage promastigotes of *L. infantum*, with an IC_50_ of 177.9 µM. Nevertheless, this value is much higher than that observed against intramacrophagic amastigotes. Such stage-specific effect of EGCG is not uncommon (Vermeersch et al., 2009; De Muylder et al., 2011; Gervazoni et al., 2018). Promastigotes and amastigotes differ in terms of protein expression (Holzer et al., 2006) and metabolism (Saunders et al., 2014; Jara et al., 2017) and this might translate into disparate drug susceptibilities, as observed for EGCG. Furthermore, the host cell can greatly impact drug effectiveness. This occurs with compounds that act as pro-drugs, requiring conversion to toxic derivatives by host cell factors for activity, and with molecules whose efficacy derives from their immunomodulatory properties. Pentavalent antimonials provide an example of a drug whose activity, like that of EGCG, is much more pronounced against intracellular parasites. These compounds kill parasites *via* their trivalent form, reduction apparently taking place in both the macrophage (Decuypere et al., 2005; Ponte-Sucre et al., 2017) and the amastigote, but not in promastigotes (Shaked-Mishan et al., 2001). Also contributing to parasite clearance might be their capacity to activate macrophages to produce toxic reactive oxygen and nitrogen species (Mookerjee Basu et al., 2006). Future studies will address whether any of these mechanisms applies to EGCG. Alternatively, macrophages could accumulate high levels of EGCG, as demonstrated for HIV-1 protease inhibitors which also require lower concentrations to eliminate intracellular amastigotes compared to axenic amastigotes (Trudel et al., 2008).

These parasite forms then set the basis for us to characterize the physiological alterations imposed by EGCG on *L. infantum*. In this regard, we observed that EGCG disturbs the mitochondrial polarization of *L. infantum* promastigotes, with concomitant decrease of intracellular ATP. Importantly, we also found that EGCG triggers an H_2_O_2_ boost in these parasites, whose reversion by the H_2_O_2_-eliminating enzyme catalase neutralizes the leishmanicidal effect of the flavonoid. Since trypanothione reductase (TR) is a central molecule of the H_2_O_2_ metabolism in *Leishmania* spp., we hypothesized that the EGCG-triggered boost of H_2_O_2_ resulted from TR inhibition. Our hypothesis was validated by 1) *in silico* analyses, where we found evidence that EGCG can bind to TR with *K_i_* values spanning between 3.85 and 20.49 µM (depending on the cluster under analysis). High confidence models of EGCG/TR interaction predicted that the flavonoid could act as a competitor for the trypanothione substrate of TR by occupation of its binding pocket. Importantly, these predictions were confirmed by 2) *in vitro* kinetic assays of purified recombinant TR in the presence of EGCG, revealing a Ki of 293.9 ± 66.3 μM, this Ki value is higher when compared with other TR inhibitor such trivalent antimony (Sb^+3^), clomipramine and chalcone-based compounds (Baiocco et al., 2009a; Baiocco et al., 2009b; Jones et al., 2010; Ortalli et al., 2018). Positive correlations were observed between the inhibition of *L. infantum* promastigote proliferation and both EGCG-induced TR inhibition (Pearson correlation r = 0.9521) and increased intracellular H_2_O_2_ concentration in *L. infantum* promastigotes (Pearson correlation r = 0.9476)

Altogether, the findings reported in this manuscript add EGCG to a growing list of natural products with TR inhibitory activity (Uliassi et al., 2017; Ortalli et al., 2018). We reckon that, by interfering with this enzyme, central to the redox homeostasis of *Leishmania* spp. and related trypanosomatids, EGCG impairs the antioxidant response of the parasites, leading to the development of oxidative stress and resulting in the destruction of cellular macromolecular components promoting parasite death. This mechanism is also the basis of various antiprotozoal medications used to combat parasites in infected cells (Pal and Bandyopadhyay, 2012). At this point, we cannot exclude that EGCG also blocks other *Leishmania* molecules, being the accumulated inhibitory effect of the flavonoid over different targets that leads to parasite death.

In conclusion, the present data provide further insights into the mechanisms of action of EGCG against *Leishmania infantum*. Our results strongly suggest that EGCG, an effective compound for the treatment of visceral leishmaniasis (Inacio et al., 2019), competitively inhibits TR activity, causing an oxidative imbalance that leads to a decrease in mitochondrial membrane potential, thus reducing the intracellular ATP concentration.

## Data Availability Statement

The original contributions presented in the study are included in the article/**Supplementary Material**. Further inquiries can be directed to the corresponding author.

## Ethics Statement

The animal study was reviewed and approved by Committee on the Ethics of Animal Experiments of the Instituto Oswaldo Cruz (CEUA-IOC, License Number: L-11/2017).

## Author Contributions

Conceptualization: JI and EA-A. Data curation: JI, MF, GL-S, HC, and EA-A. Formal analysis: JI, MF, GL-S, AT, HC, and EA-A. Investigation: JI, GL-S, AT, HC, and EA-A. Methodology: JI, MF, GL-S, AT, HC, and EA-A. Writing – original draft: JI, AT, HC, and EA-A. Writing – review and editing, JI, GL-S, AT, HC, and EA-A. All authors contributed to the article and approved the submitted version.

## Funding

This work was supported by Fundação Carlos Chagas Filho de Amparo a Pesquisa do Estado do Rio de Janeiro (FAPERJ), Conselho Nacional de Desenvolvimento Científico e Tecnológico (CNPq), Programa Estratégico de Apoio a Pesquisa em Saúde (PAPES/FIOCRUZ);, and Fundação Oswaldo Cruz (FIOCRUZ). EA-A is the recipient of a research scholarship from Conselho Nacional de Desenvolvimento Científico e Tecnológico (CNPq). JI was supported by a postdoctoral fellowship from Conselho Nacional de Desenvolvimento Científico e Tecnológico (CNPq). JI is a recipient of a postdoctoral fellowship from Fundação Oswaldo Cruz (FIOCRUZ). HC is funded by Fundação para a Ciência e Tecnologia (FCT, Portugal) through the “Investigador FCT” contract IF/01244/2015.

## Conflict of Interest

The authors declare that the research was conducted in the absence of any commercial or financial relationships that could be construed as a potential conflict of interest.
