# Supplementary figures and images for: Epigallocathechin-O-3-Gallate Inhibits Trypanothione Reductase of Leishmania infantum, Causing Alterations in Redox Balance and Leading to Parasite Death
Source: Front Cell Infect Microbiol. 2021 Mar 25;11:640561. doi: 10.3389/fcimb.2021.640561 (PMC8027256; doi:10.3389/fcimb.2021.640561)

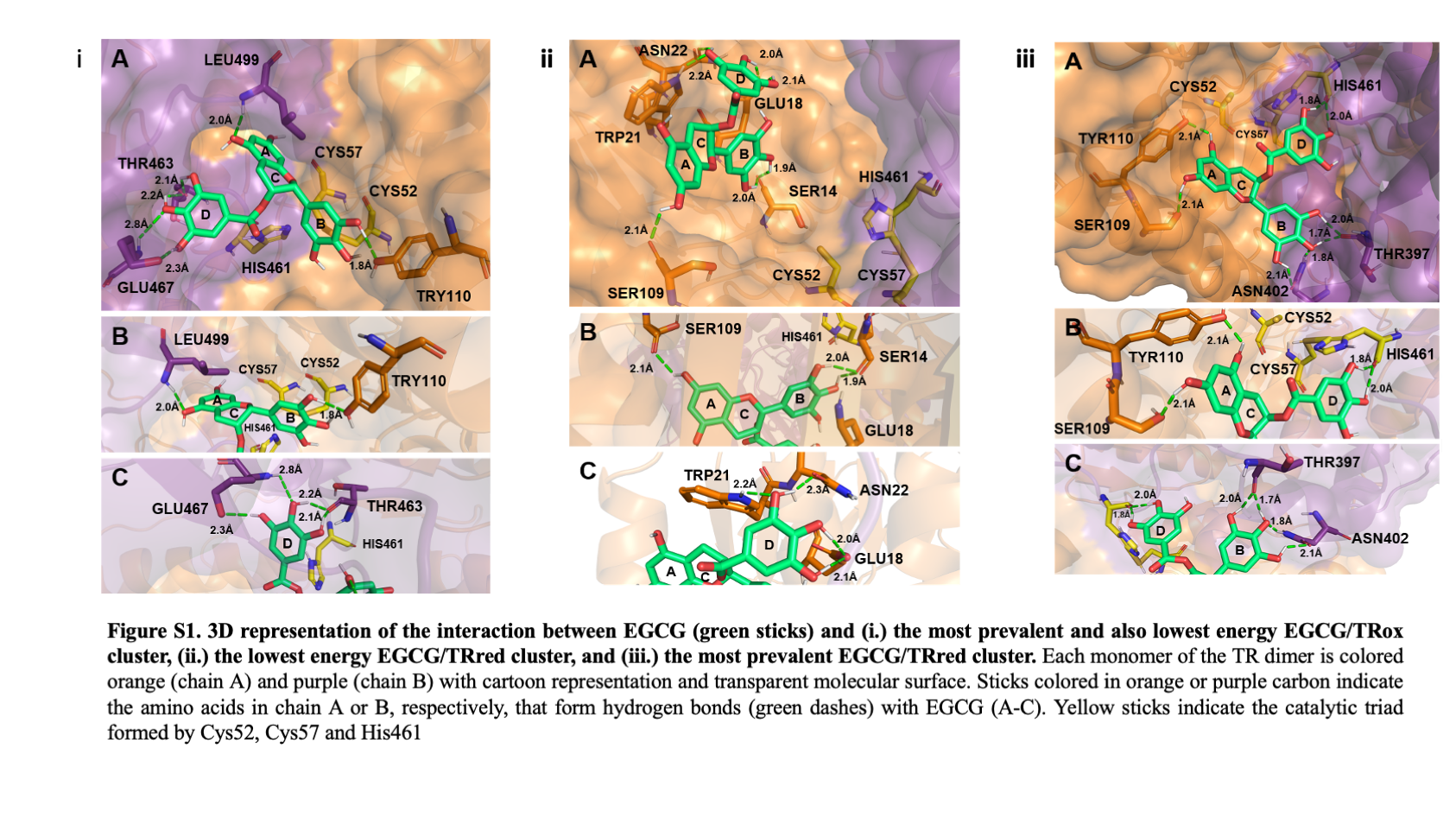

Supplement: Supplementary file 1 [file Image_1.tif]

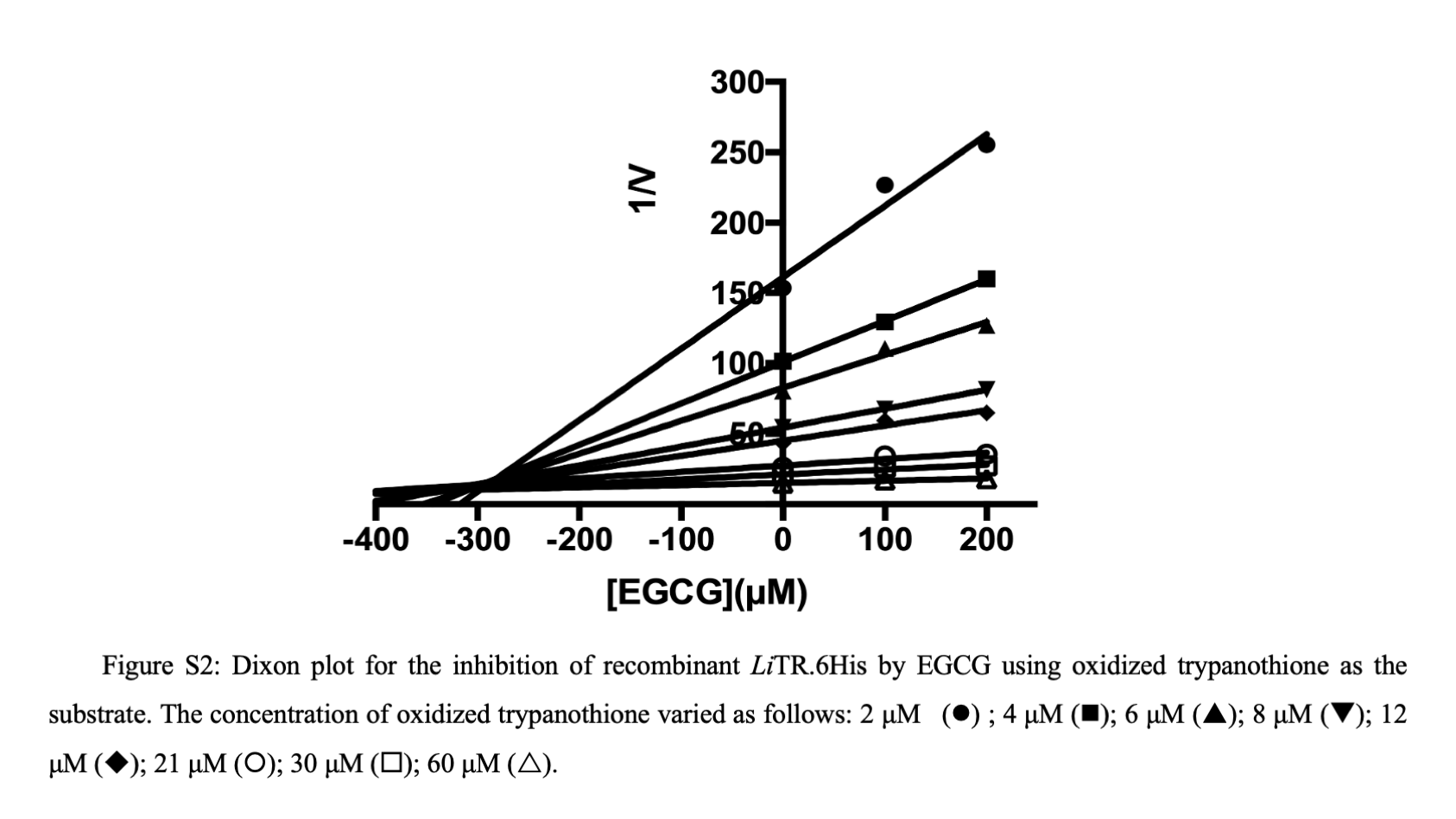

Supplement: Supplementary file 2 [file Image_2.tif]
